# Neuroplasticity of peripheral axonal properties after ischemic stroke

**DOI:** 10.1371/journal.pone.0275450

**Published:** 2022-10-04

**Authors:** Hung-Ju Chen, Jowy Tani, Cindy Shin-Yi Lin, Tsui-San Chang, Yi-Chen Lin, Ting-Wei Hsu, Jia-Ying Sung

**Affiliations:** 1 Department of Neurology, Wan Fang Hospital, Taipei Medical University, Taipei, Taiwan; 2 Taipei Neuroscience Institute, Taipei Medical University, Taipei, Taiwan; 3 Department of Neurology, School of Medicine, College of Medicine, Taipei Medical University, Taipei, Taiwan; 4 Neural Regenerative Medicine, College of Medical Science and Technology, Taipei Medical University and National Health Research Institutes, Taipei, Taiwan; 5 Translational Research Collective, Faculty of Medicine and Health, Brain & Mind Centre, The University of Sydney, Sydney, Australia; 6 Department of Nursing, Chang Gung University of Science and Technology, Taipei, Taiwan; University of Texas Health Science Center, UNITED STATES

## Abstract

**Objective:**

This study investigated how peripheral axonal excitability changes in ischemic stroke patients with hemiparesis or hemiplegia, reflecting the plasticity of motor axons due to corticospinal tract alterations along the poststroke stage.

**Methods:**

Each subject received a clinical evaluation, nerve conduction study, and nerve excitability test. Nerve excitability tests were performed on motor median nerves in paretic and non-paretic limbs in the acute stage of stroke. Control nerve excitability test data were obtained from age-matched control subjects. Some patients underwent excitability examinations several times in subacute or chronic stages.

**Results:**

A total of thirty patients with acute ischemic stroke were enrolled. Eight patients were excluded due to severe entrapment neuropathy in the median nerve. The threshold current for 50% compound muscle action potential (CMAP) was higher in paretic limbs than in control subjects. Furthermore, in the cohort with severe patients (muscle power ≤ 3/5 in affected hands), increased threshold current for 50% CMAP and reduced subexcitability were noted in affected limbs than in unaffected limbs. In addition, in the subsequent study of those severe patients, threshold electrotonus increased in the hyperpolarization direction: TEh (100–109 ms), and the minimum I/V slope decreased. The above findings suggest the less excitable and less accommodation in lower motor axons in the paretic limb caused by ischemic stroke.

**Conclusion:**

Upper motor neuron injury after stroke can alter nerve excitability in lower motor neurons, and the changes are more obvious in severely paretic limbs. The accommodative changes of axons progress from the subacute to the chronic stage after stroke. Further investigation is necessary to explore the downstream effects of an upper motor neuron insult in the peripheral nerve system.

## Introduction

Stroke is regarded as a disease involving damage to the central nervous system; however, persistent changes can occur in lower motor neurons when central pathways are disrupted by stroke [1,2]. The nerve excitability test uses threshold tracking techniques to study axon excitability and neuronal plasticity in vivo by indirectly examining ion channels and the resting membrane potential [3,4]. Automated protocols for assessing nerve excitability have been used in numerous studies, investigating the pathophysiology of diseases affecting central to peripheral nerve systems, such as spinal cord injury, cerebellar disorders, amyotrophic lateral sclerosis, radiculopathy, fibromyalgia, and metabolic, toxic, and demyelinating neuropathies [5–12]. Excitability properties of the median nerve have been studied in people with ischemic and hemorrhagic stroke. Inward rectifier (*I*_H_) is one of the voltage-dependent ion conductance located on axons, which is activated by hyperpolarization. Changes in *I*_H_ indicate higher axonal thresholds in the paretic limb (P) than in the non-paretic limb (NP) during 100 ms hyperpolarizing currents, and a tendency for the paretic limb axons to express less *I*_H_ was found [13–15]. However, there is a difference in changes in nerve excitability between acute and chronic stages. First, in chronic stroke patients, more prominent changes in several different parameters of nerve excitability studies were noted [15], while Huynh et al. reported no significant changes in nerve properties between the acute stage and the follow-up three months later [13]. Thus, additional data is needed to evaluate the axonal properties in the acute poststroke stage which have been less discussed in previous studies, and whether those properties change along the time. Second, it was reported that reduced *I*_H_ is correlated with a reduction in maximal voluntary muscle contraction in chronic stroke patients [15], but results of nerve excitability studies in acute stroke patients seem not correlated with the extent of muscular weakness or the clinical recovery. Huynh and colleagues demonstrated the accommodation changes in affected hands in a longitudinal stroke study; however, whether these changes were related to the mobility of affected hands was not mentioned [13]. This study aimed to investigate the association of peripheral neuroplasticity and ischemic stroke-related motor impairment and elucidate the clinical significance of altered nerve excitability in poststroke stages.

## Materials and methods

Patients with hemiparesis attributed to a first-time acute ischemic stroke were prospectively recruited from Wan Fang Hospital, Taipei Medical University, Taipei, Taiwan. Subjects with symptoms suggestive of peripheral nerve dysfunction or taking medications that would influence nerve excitability parameters were excluded. Patients with prominent edema on the affected side or significant entrapment peripheral neuropathy in the upper limbs revealed by a conventional nerve conduction study were also excluded from the study. According to our laboratory references of nerve conduction studies, we defined mononeuropathy of the median nerve at the wrist when the distal latency was longer than 4.8 ms or the difference of sensory nerve conduction velocity (NCV) between the index and the fourth fingers was greater than 0.4 m/sec.

Infarction was diagnosed when brain magnetic resonance imaging (MRI) showed acute lesions corresponding to clinical deficits. MRI was performed within seven days of onset, usually defined as the acute stage of ischemic stroke for obtaining an exact location of infarcts. After enrollment, each patient underwent a neurological examination. The severity of stroke was assessed by the National Institute of Health Stroke Scale (NIHSS, 0–42 with 42 corresponding to the highest severity). Muscle strength was recorded by the Medical Research Council scale (MRC, 0–5 with 0 corresponding to the weakest muscle). Additionally, the modified Rankin Scale (mRS, 0–6 with 0 corresponding to no symptom or disability) was used to measure the degree of dependence in daily activities. Laboratory data, including serum fasting glucose, HbA1c, renal function, and liver function tests, were recorded. Patients received nerve excitability tests on both the paretic and non-paretic sides in the acute stage. Control nerve excitability test data were obtained from twenty-two age- and sex-matched control subjects who did not have known neurological disorders or abnormal neurological examination findings. Follow-up tests were performed in patients during subacute or chronic stages. Written informed consent was obtained from all subjects, and some consents were obtained from a legally authorized surrogate due to difficulty in writing caused by stroke. The study was approved by the Joint Institution Review Board of Taipei Medical University.

### Nerve excitability tests

Nerve excitability studies were performed on bilateral median nerves with compound muscle action potentials (CMAPs) recorded from the abductor pollicis brevis muscles according to previously described protocols [16,17]. Median nerves of paretic and nonparetic hands were stimulated at the wrists by different stimulation strength outputs from the isolation bipolar current stimulator (Digitimier DS5 stimulator), and the stimulation current and recording of threshold changes were controlled by QTRAC software (Institute of Neurology, London, UK). The skin temperature was maintained above 32°C. Information on the axonal membrane and ion channel properties was obtained indirectly by conditioned protocols [17–21]. The target response in the stimulus-response curve was set at the steepest point, and changes in the threshold of the stimulus current (mA) were tracked. Rheobase (mA) in the strength-duration relationship represents the minimal current required to produce an action potential when the stimulus is infinitely long. The strength–duration time constant (SDTC) (msec) was estimated by Weiss’ equation from thresholds in test stimuli of different durations [22]. The current-threshold *(I/V)* relationship was assessed by the threshold change at the end of 200-ms polarizing currents. The threshold electrotonus was measured using subthreshold 100-ms polarizing currents in both depolarizing (TEd; +40%) and hyperpolarizing (TEh; -40%) directions to alter potentials across the internodal membrane. The recovery cycle used a supramaximal conditioning stimulus followed by tracking the thresholds at interstimulus intervals from 2 to 200 ms. The recovery cycle consisted of a relative refractory period (RRP), a superexcitable period, and a late subexcitable period. Superexcitability was measured as the maximal threshold reduction, and subexcitability was measured as the maximal threshold increase after an interstimulus interval of 10 ms [16–18,23].

### Statistical analysis

All statistical analyses were performed with the packaged software SPSS version 19.0 for Windows (SPSS Inc., Chicago, U.S.A.). The unpaired t-test was used to analyze differences in nerve excitability parameters between the patients with ischemic stroke and control data. Comparison of the nerve excitability test and nerve conduction study parameters in the paretic versus the non-paretic limb, and the acute versus the follow-up test in each subject was performed by using the paired t-test. Differences were considered statistically significant at values of p < 0.05.

## Results

### Clinical profiles of patients

Thirty-one patients were recruited initially in this study. One patient refused the nerve excitability test, and eight patients were excluded because of prominent median entrapment neuropathy or old injuries in areas to be sampled. A total of 22 patients (10 men, 12 women; age range, 43–86 years; mean, 66.08±12.85 years) received examinations according to the study protocol within the acute period (mean, 5.5 days). The paretic limbs were on the right side in 13 patients and on the left side in 9 patients. Two patients had large infarcts in middle cerebral artery territories, one had pontine infarct, and the rest had infarcts involving subcortical and ganglionic levels. The mean NIHSS score was 5.7 (range, 0–21), and the mean mRS score was 2.9 (range, 0–5) (Table 1). Four patients had diabetes mellitus, and all controls had not been diagnosed with diabetes mellitus. There were no significant differences in parameters for the nerve conduction study on median nerves between paretic and non-paretic limbs (latency 4.0±0.48 ms in P, 4.0±0.54 ms in NP; amplitude 6.2±2.2 mV in P, 6.8±1.79 mV in NP; NCV 51±6.13ms in P, 52±4.94 ms in NP).

**Table 1:** Demographic and clinical profiles.

No.	Age, years	Sex	Lesion site _a_	Side _b_	TSO _c_	MRC _d_	NIHSS _e_	mRS _f_
1	64	Female	CR,IC	R	2	4+	2	2
3	66	Female	LN	L	6	4+	0	1
6	75	Male	CR,IC	R	5	4	2	2
7	64	Female	Frontoparietal C, CR	R	5	2	13	4
9	86	Male	CR	R	2	3	5	4
11	77	Female	CR,EC,LN	L	6	1	21	5
12	50	Male	Large MCA territory	L	5	0	8	3
13	77	Female	CR,IC,LN,CN	R	5	4	5	2
16	58	Male	Large MCA territory	R	5	0	16	5
17	67	Female	CR,BG	R	8	1	8	5
18	85	Female	LN,IC,CN	R	7	4+	1	1
20	58	Male	LN, C	L	5	4	4	2
22	43	Male	pons	L	5	4	3	2
23	69	Female	CR,IC,LN	R	9	0	7	4
24	55	Female	CR	L	4	4+	2	2
25	56	Male	CN,CR,LN	L	5	2	7	4
26	79	Female	LN,IC,CR	L	6	4	3	3
27	78	Female	IC,CR	L	5	4	3	3
28	73	Male	CR,IC,LN,EC	R	7	2	6	4
29	56	Female	LN,CR,CN,EC	R	6	4+	4	2
30	57	Male	IC, thalamus	R	5	3	6	3
31	56	Male	CR,IC	R	6	4	1	1

_a_ Lesion site, *IC* internal capsule, *EC* external capsule, *CR* corona radiata, *LN* lentiform neuclei, *CN* caudate neuclei, *C* cortical.

_b_ Side of paretic limb, *R* right side, *L* left side.

_c_ TSO, days since onset of stroke.

_d_ MRC, muscle power of paretic hand.

_e_ *NIHSS* National Institutes of Health Stroke Scale.

_f_ *mRS* modified Rankin Scale.

### Motor nerve excitability profiles

**Fig 1:**
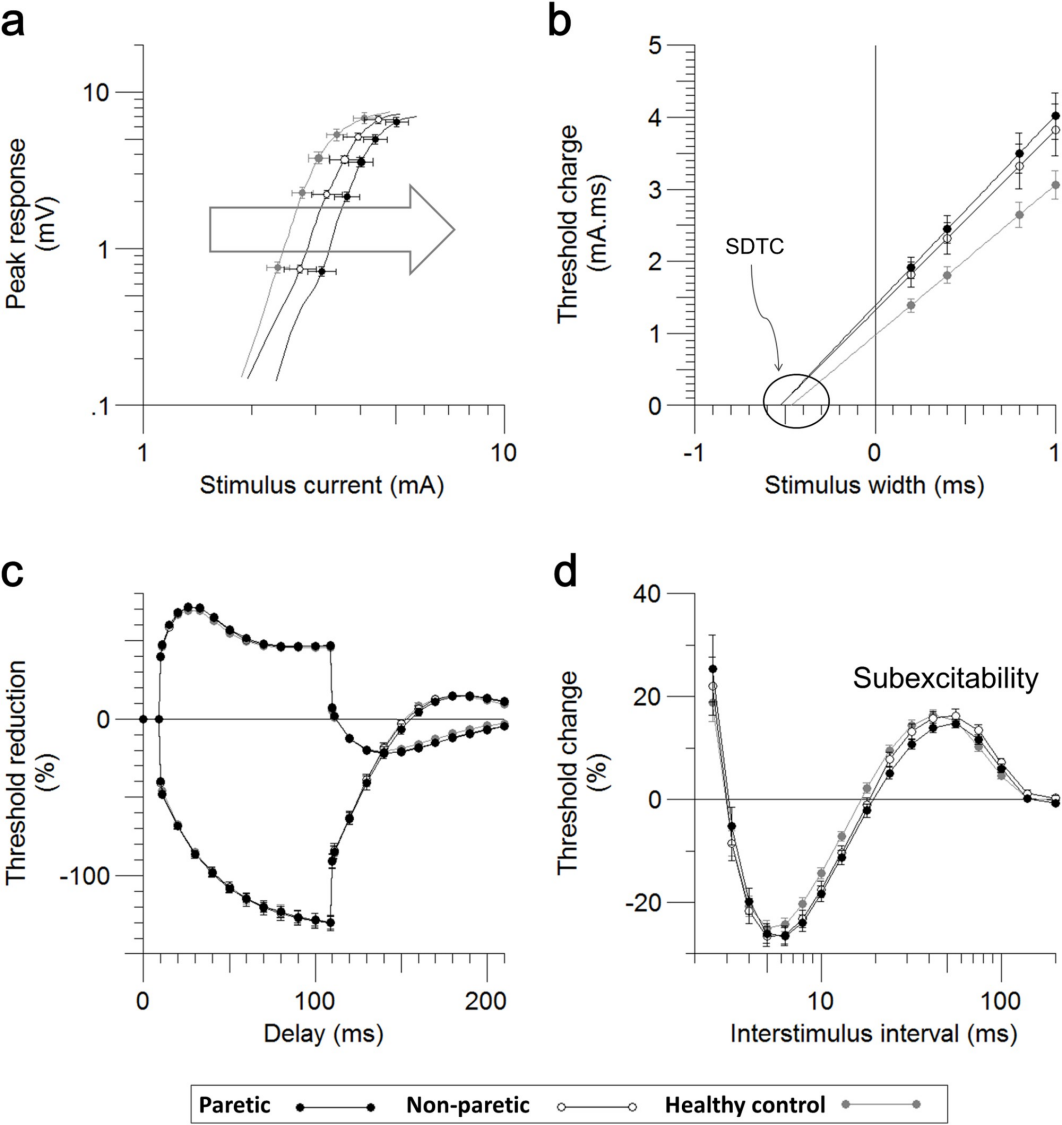
The waveforms of motor axons excitability parameters were presented as mean with SE for the paretic (black, n = 22), non-paretic (white, n = 22) limbs, and the sex and age-matched controls (grey, n = 22). **a.** Stimulus current for 50% maximal response is higher in paretic limbs than in non-paretic limbs and controls. **b.** SDTC and rheobase current present significant differences between paretic limbs and controls. **c.** No differences in the threshold reduction among paretic, non-paretic limbs and controls either in depolarizing or hyperpolarizing conditioning-current stimulation in the acute stage. **d.** Subexcitability was smaller in the paretic limbs than in non-paretic limbs.

The stimulus current for 50% maximal response in the stimulus-response curve was higher in paretic limbs than in non-paretic limbs and controls (4.26±0.31 mA in P, 3.9±0.38 mA in NP, 3.20±0.21 in controls), but the difference was only significant between paretic limbs and controls (*P* = 0.007) (Table 2, Fig 1A). There was also a significant difference in SDTC (0.54±0.02 ms in P, 0.47±0.02 ms in control, *P* = 0.036) and the rheobase current (2.64±0.22 mA in P, 2.09±0.14 mA in control, *P* = 0.042) between paretic and control subjects (Table 2, Fig 1B), but there was no significant difference between paretic and non-paretic limbs. In threshold electrotonus, there was no significant change in the threshold reduction among paretic, non-paretic, and control subjects in either depolarizing or hyperpolarizing conditioning-current stimulation in the acute stage. In measurements of threshold electrotonus, TEd with specified time in parentheses means thresholds at the specified time during depolarizing threshold electrotonus, TEh with specified time in parentheses means threshold at the specified time during hyperpolarizing threshold electrotonus, and S2 accommodation indicates the difference between the peak threshold and threshold at 100 ms (Table 2, Fig 1C). In the recovery cycle, the subexcitability was significantly lower in the paretic limbs than in the non-paretic limbs (14.09±0.81% in P, 16.16±1.18% in NP, *P* = 0.027), while the superexcitability was not different (-26.15±1.68% in P, -26.59±1.68% in NP, *P* = 0.53) (Table 2, Fig 1D).

**Table 2:** Comparison of axonal properties by the excitability test in paretic (P), non-paretic (NP), and control (C) limbs.

	Paretic Mean ± SE			Non-paretic Mean ± SE			Control Mean ± SE			P vs NP P-value	P vs C P-value	NP vs C P-value
Stimulus response												
CMAP peak, mV	7.51	±	0.47	7.65	±	0.39	8.17	±	0.63	0.690	0.404	0.480
Stimulus for 50% CMAP, mV	4.26	±	0.31	3.90	±	0.38	3.20	±	0.21	0.203	0.007**	0.120
Stimulus-response slope	4.46	±	0.27	4.37	±	0.29	4.04	±	0.25	0.745	0.267	0.399
Stimulus width-charge												
SDTC, ms	0.54	±	0.02	0.53	±	0.02	0.47	±	0.02	0.755	0.036*	0.063
Rheobase, mA	2.64	±	0.22	2.47	±	0.25	2.09	±	0.14	0.378	0.042*	0.196
TE to ±40% currents												
TEd(10–20 ms), %	70.21	±	1.18	70.15	±	0.98	68.42	±	1.10	0.943	0.274	0.247
TEd(90–100 ms), %	46.80	±	1.28	46.61	±	1.19	45.54	±	0.93	0.789	0.430	0.482
TEd (undershoot), %	-21.49	±	0.81	-21.38	±	0.99	-20.15	±	0.91	0.907	0.274	0.363
S2 accommodation, %	23.03	±	0.87	23.19	±	0.84	22.42	±	0.98	0.838	0.638	0.550
Accommodation 1⁄2time,ms	39.59	±	1.07	39.15	±	1.09	38.21	±	0.90	0.617	0.331	0.509
TEh (90-100ms), %	-129.2	±	4.06	-129.68	±	4.69	-129.1	±	4.63	0.872	0.987	0.931
TEh (100-109ms), %	-129.2	±	4.06	-129.7	±	4.69	-129.1	±	4.63	0.877	0.987	0.933
TEh (overshoot), %	15.17	±	1.09	15.37	±	0.95	15.12	±	0.93	0.834	0.974	0.850
Recovery cycle												
RRP, ms	3.17	±	0.13	3.09	±	0.13	3.02	±	0.11	0.449	0.388	0.657
Refractoriness at 2.5 ms, %	25.43	±	6.49	22.00	±	5.67	18.78	±	3.60	0.274	0.364	0.627
Superexcitability, %	-26.15	±	1.68	-26.59	±	1.68	-24.28	±	1.24	0.531	0.376	0.275
Subexcitability, %	14.09	±	0.81	16.16	±	1.18	15.57	±	0.93	0.027*	0.236	0.697
I/V relationship												
Resting IV slope	0.52	±	0.19	0.53	±	0.20	0.58	±	0.02	0.849	0.061	0.117
Minimum IV slope	0.25	±	0.02	0.25	±	0.01	0.24	±	0.01	0.766	0.673	0.630
Hyperpol. IV slope	0.51	±	0.06	0.42	±	0.04	0.44	±	0.04	0.096	0.327	0.639
Temperature, °C	34.38	±	0.26	34.68	±	0.24	34.36	±	0.17	0.154	0.942	0.251

*CMAP* Compound Muscle Action Potential, *SDTC* strength-duration time constant, *RRP* relative refractory period

* p<0.05

** p<0.01.

### Nerve excitability changes in patients with severe weakness

**Fig 2:**
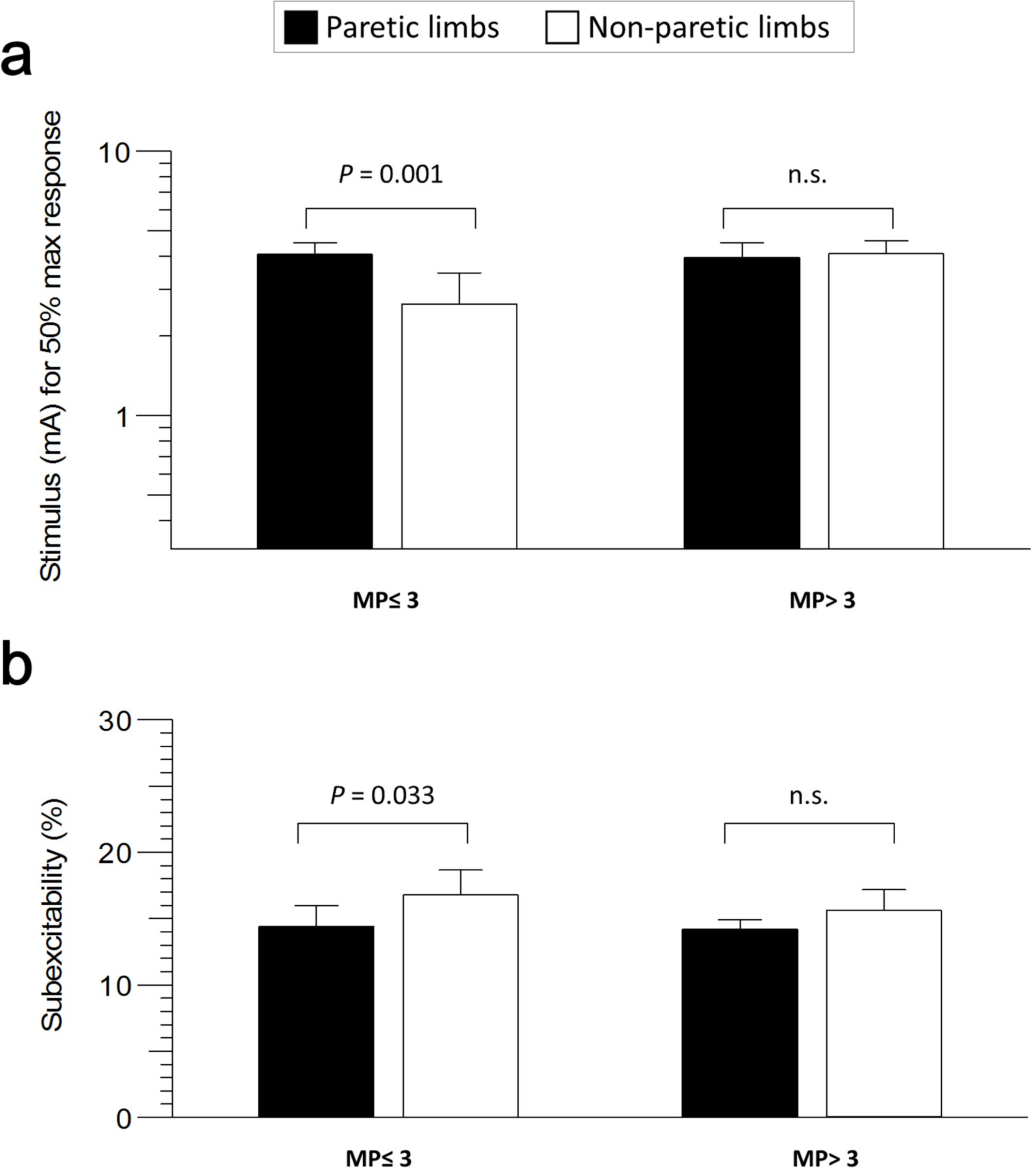
Comparison of the different nerve excitability changes between paretic limbs (black bars) and non-paretic limbs (white bars) in the patients with severe (MP≤3) and mild (MP>3) weakness. **a.** Increased stimulus current needs to achieve 50% maximal response of paretic limbs in the MP≤ 3, but no change in the MP> 3 cohort. **b.** Decreased subexcitability of paretic limbs only showed significantly in the MP≤ 3 cohort.

Subjects were divided into two cohorts based on the grading of muscle power (MP) in paretic limbs. Severe weakness was defined as MP ≤ 3, and mild weakness was MP > 3. The characteristics between these two cohorts, including age (66.08±12.85 years in MP > 3, 65.70±10.98 years in MP ≤ 3), test time since onset (5.17±1.27 days in MP > 3, 5.7±1.95 days in MP ≤ 3), and the HbA1c (6.5±1.62% in MP > 3, 5.9±1.02% in MP ≤ 3) were similar. The NIHSS score was significantly lower in the MP > 3 cohort than in the MP ≤ 3 cohort (2.5±1.45 in MP > 3, 9.7±5.25 in MP ≤ 3, *P*< 0.001) (Table 3). In the MP ≤ 3 cohort, the greater stimulus current for 50% maximal response in the stimulus-response curve and smaller subexcitability in paretic limbs than in non-paretic limbs were statistically significant (stimulus-response, 4.27±1.49 mA in P, 3.3±1.77 mA in NP, *P* = 0.001; subexcitability, 14.39±5.05% in P, 16.78±5.94% in NP, *P* = 0.033) (Fig 2A and 2B). In contrast, there was no significant difference in all measurements of the MP > 3 cohort.

**Table 3:** Clinical profiles of subgroups by the muscle strength in paretic limbs.

	MP≤ 3	MP>3
Male/ female	6 / 4	4 / 8
Side of paresis (Right/Left)	7 / 3	6 / 6
Age, years	65.70(10.98)	66.08(12.85)
Duration of onset, days	5.7(1.95)	5.17(1.27)
NIHSS*	9.7(5.25)	2.5(1.45)
HbA1c	5.9(1.02)	6.5(1.62)

Categorical data were summarized as counts; continuous measures were summarized in mean with standard deviation in parentheses.

* Significant difference between cohorts in NIHSS (P< 0.001).

### Longitudinal follow-up nerve excitability for patients

**Fig 3:**
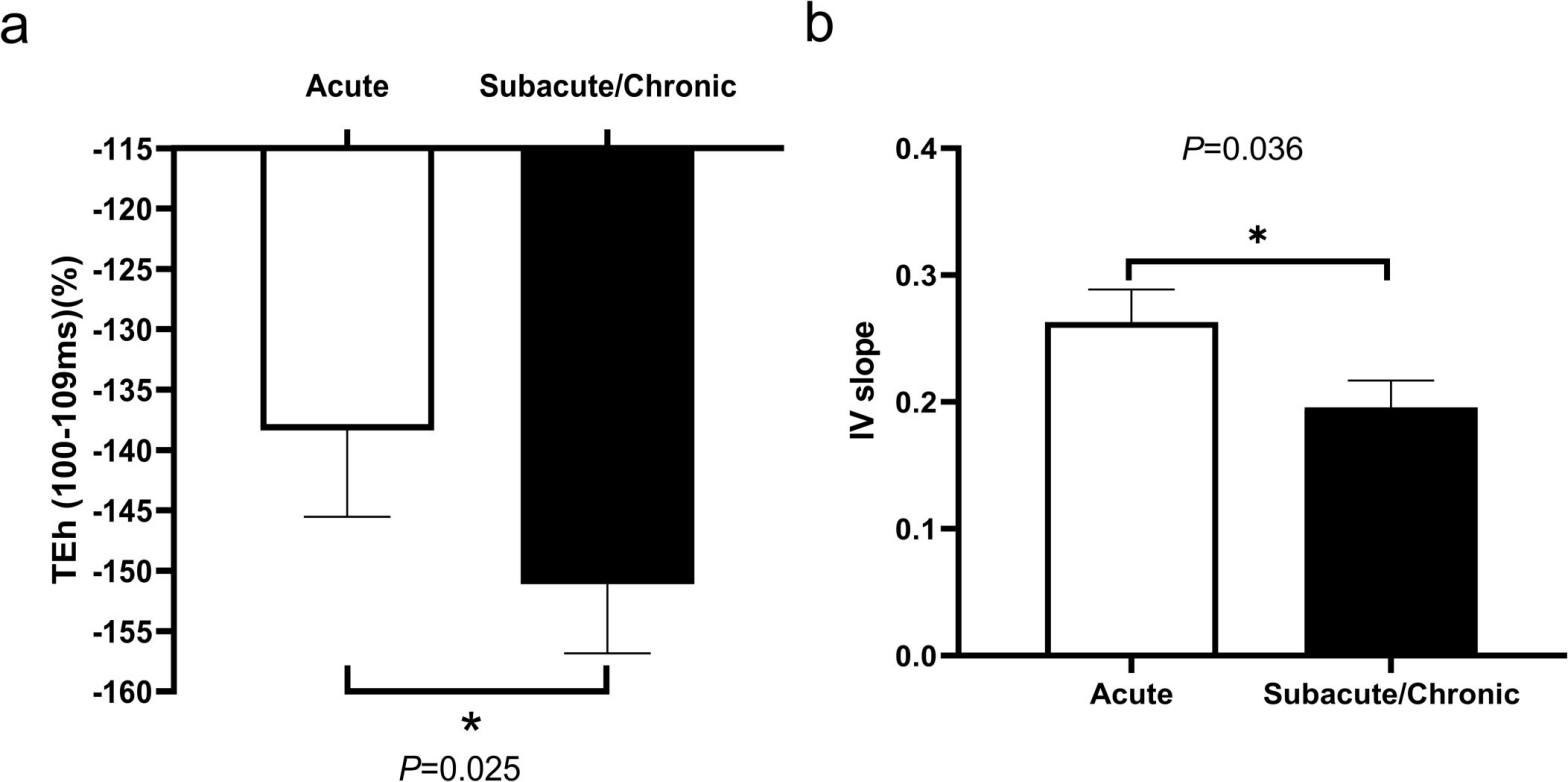
Comparison of follow-up tests (black bars) to the acute stage ones (white bars) in threshold electrotonus change and current-threshold (I/V) relationship. **a.** There was a greater change in the threshold at 100-109ms during hyperpolarizing threshold electrotonus (TEh)*P = 0.025. **b.** The minimum I/V slopes in the hyperpolarized direction became smaller in follow-up studies (*P* = 0.036).

Six subjects in the MP ≤ 3 cohort received follow-up nerve excitability tests (duration: 17–873 days, mean 360.62 days), and a total of eight paired tests showed that follow-up threshold electrotonus under the hyperpolarizing conditioning current, TEh (100–109 ms), was significantly greater than the one in the acute stage (acute: -138.36±7.15%, follow-up: -151.09±5.74%, *P* = 0.025) (Fig 3A). The minimum I/V slopes in the hyperpolarized direction were also smaller in the follow-up study (prior: 0.26±0.03, follow-up: 0.20±0.03, *P* = 0.036) (Fig 3B). Other parameters did not change significantly between tests, and there was no significant change in excitability properties of non-paretic limbs during the follow-up period.

## Discussion

### Acute changes in axonal excitability in paretic limbs indicate nodal inactivation

Chronic upper motor neuron damage can induce corticospinal tract degeneration and it has been seen in neuroimaging studies [24]. The corresponding biophysiological changes in the peripheral axon are still unclear. The results of this study showed the peripheral axonal activity in affected limbs was less excitable after central nervous system insults, i.e., increased stimulus current for 50% CMAP. In addition, SDTC and the rheobase current were greater in the paretic limbs than in the control limbs. SDTC and the rheobase current are determined by persistent sodium channel function [18]. These results may indicate that peripheral axons become more difficult to activate after acute stroke. Turan and Zinnuroglu reported axonal excitability in subacute stroke. Their results demonstrated significant changes in the peak response, refractoriness, subexcitability, and TEd20 in affected limbs [25]. It is compatible with nodal inexcitability. Our results showed changes in SDTC and rheobase current. However, the findings of both studies support that axons become relatively inexcitable in association with nodal dysfunction in paretic limbs.

In our study, a change in subexcitability was also observed in the acute stroke stage. Reduced subexcitability was more obvious in patients with severely weak limbs. A flattened recovery cycle related to the reduction in both subexcitability and superexcitability was also reported in the chronic stage after stroke [15]. Subexcitability is one of the most sensitive parameters in membrane potential changes [26]. One possible explanation is that axons become in-excitable in acute stroke, and then nodal and intermodal axons are difficult to be charged. Consequently, slow potassium conductance decreases and results in reduced subexcitability. The process might be related to downstream regulation from central nervous system lesions.

### Longitudinal declination of accommodation indicates neural plasticity in peripheral axons after stroke

Threshold electrotonus, determined by membrane potential, internodal conductance, and myelin thickness, is a common index of axon accommodation [4,27]. The threshold current changes in depolarization and hyperpolarization are associated with voltage-dependent ion conductance, especially potassium and the inward rectifier (*I*_H_), which is located mainly in the internode and activated by hyperpolarization and permeable to both potassium and calcium conductance, and sensitive to the cyclicadenosine monophosphate (cAMP) level [28]. In a prolonged hyperpolarizing membrane, *I*_H_ will be activated to decrease the threshold current change, i.e., limit the changes of membrane potential. In our study, TEh (100–109 ms) in the paretic limb did not change in the acute stage, but it became significantly greater over time. The results showed that paretic limb axons have a progressively reduced capacity to accommodate hyperpolarizing currents due to downregulation of *I*_H._ Reduced HCN channel expression was reported in corticospinal lesions [14,28]. Klein et al. reported that reduced *I*_H_ is correlated with a reduction in maximal voluntary muscle contraction in chronic stroke patients [15]. The significant difference of current-threshold relationship with reduced minimum I/V slope in the follow-up test supports the viewpoint of the evolution of internodal conductance.

The reduced accommodation might be related to axon hyperpolarization after upper motor neurons are injured [8,29]. The change is considered to be a consequence of impairment of supraspinal control [30] and reduced metabolism in axons [31]. The alterations in the conductance of the inward rectifier and slow potassium are also probably in association with hyperpolarization in motor axons linked to proximal lesions [32]. Distal nerve axonal hyperpolarization related to Na^+^-K^+^ ATPase overactivation induced by proximal ischemia was also noted in patients with cervical radiculopathy [8]. In ischemic stroke, the long tracts of the motor system are damaged proximally to the paretic limbs, producing prolonged changes and adaptation in downstream neurotransmission, which cause so-called neural plasticity and thereby affect motor axonal properties and their response to stimuli. Spinal cord injury, another upper motor neuron disease, can also contribute to motor axon property changes in the peripheral nervous system [5].

The changes in threshold electrotonus and current-threshold relationship corresponded to acute or chronic poststroke stages; thus, these parameters can be possibly used as indicators in studies that focus on the timing of interventions. For example, very early mobilization was reported effective in improving the functional status following acute stroke [33]. TEh(100–109 ms) and I/V slope might serve to understand the early mobilization effect on peripheral axons after stroke in the future study.

### Association of altered axonal excitability and clinical manifestations

In the present study, the extent of changes in axonal properties seemed associated with the severity of weakness according to the results of subgroup analysis. We suppose the alternation in axonal properties is too subtle to detect in a limb with mild weakness. Klein et al. reported that lower *I*_H_ and Na^+^ conductance might contribute to lesser neuromuscular activation in stroke patients [15]. Inwardly-rectifying potassium (K_IR_) channels can facilitate rapid vasodilatation during exercise with an increasing intensity, and mediate augmentation of blood flow when greater muscle fiber recruitment is required [34]. Hence, we assume that if inward rectifier can be enhanced, there will be a higher chance for better recovery in stroke related motor impairment. The mobilization of affected limbs may be related to the maintenance of the normal accommodation of peripheral axons. Peripheral stimulation was documented to be beneficial to facilitate the effect of central stimulation in stroke patients, and consequently attain improvement in motor function [35]. Nerve excitability test could be a feasible and simple tool for assessing the effect or exploring the mechanism. More studies are necessary to prove how the axonal properties change after peripheral stimulation applied to patients with stroke.

In conclusion, peripheral axonal properties, including modulation of *I*_H_ and potassium channel conductance, alter to different extents according to the severity of a paretic limb, starting in the acute stroke phase. Progression of those changes in severely affected limbs might result in lesser accommodation. Neuroplasticity after ischemic stroke might be associated with the evolution of axonal excitability properties, and further investigation is needed to determine the biophysiological interaction between upper and lower motor neurons.

### Limitations and future perspectives

The sample size is small, and only a few subjects underwent follow-up nerve excitability tests. In addition, there is a lack of information about patients with mild weakness in their chronic stages. To compare and analyze the peripheral axonal property in stroke patients with good or poor motor recovery should be conducted to know whether it can be a prognostic biomarker of stroke. Studies focusing on the impact of interventions on both central and peripheral nervous systems after stroke might provide essential predictive information and improve the effectiveness of poststroke management.
